# Combination of triapine, olaparib, and cediranib suppresses progression of BRCA-wild type and PARP inhibitor-resistant epithelial ovarian cancer

**DOI:** 10.1371/journal.pone.0207399

**Published:** 2018-11-16

**Authors:** Z. Ping Lin, Yong-Lian Zhu, Ying-Chun Lo, Jake Moscarelli, Amy Xiong, Yasmin Korayem, Pamela H. Huang, Smith Giri, Patricia LoRusso, Elena S. Ratner

**Affiliations:** 1 Department of Obstetrics, Gynecology, and Reproductive Sciences, Yale University School of Medicine, New Haven, Connecticut, United States of America; 2 Department of Pathology, Yale University School of Medicine, New Haven, Connecticut, United States of America; 3 Section of Medical Oncology, Yale Cancer Center, Yale University School of Medicine, New Haven, Connecticut, United States of America; University of South Alabama Mitchell Cancer Institute, UNITED STATES

## Abstract

PARP inhibitors target BRCA mutations and defective homologous recombination repair (HRR) for the treatment of epithelial ovarian cancer (EOC). However, the treatment of HRR-proficient EOC with PARP inhibitors remains challenging. The objective of this study was to determine whether the combination of triapine (ribonucleotide reductase inhibitor), cediranib (vascular endothelial growth factor receptor tyrosine kinase inhibitor), and the PARP inhibitor olaparib synergized against BRCA wild-type and HRR-proficient EOC in xenograft mouse models. In addition, the mechanisms by which cediranib augmented the efficacy of triapine and olaparib were investigated. BRCA-wild type and PARP inhibitor-resistant EOC cell lines were implanted subcutaneously (s.c.) into nude mice or injected intraperitoneally (i.p.) into SCID-Beige mice. Mice were then treated i.p. with olaparib, cediranib, triapine, various double and triple combinations. The volume of s.c tumor in nude mice and the abdominal circumference of SCID-Beige mice were measured to evaluate the effectiveness of the treatment to delay tumor growth and prolong the survival time of mice. In both xenograft mouse models, the combination of triapine, olaparib and cediranib resulted in marked suppression of BRCA-wild type EOC growth and significant prolongation of the survival time of mice, with efficacy greater than any double combinations and single drugs. Furthermore, we identified that cediranib abrogated pro-survival and anti-apoptotic AKT signaling, thereby enhancing the efficacy of triapine and olaparib against BRCA-wild type EOC cells. Taken together, our results demonstrate a proof-of-principle approach and the combination regiment holds promise in treating BRCA-wild type and PARP inhibitor-resistant EOC.

## Introduction

Ovarian cancer is the leading cause of death among gynecological malignancies in the United States, with an overall 5-year survival rate of 45% and more than 14,000 women dying of the disease each year [1, 2]. The most common histological type of ovarian cancer is epithelial ovarian cancer (EOC) accounting for 90% of all casas [3]. Following optimal cytoreduction surgery, combination regimens consisting of platinum and paclitaxel are currently used as first-line chemotherapy for EOC [4, 5]. Despite a high clinical response rate of 75% with the initial therapy [6], most patients relapse and eventually develop platinum-resistant EOC, with overall response rate of 10–20% to second-line therapy [7, 8].

Olaparib (Lynparza) is the first-in-class poly ADP-ribose polymerase (PARP) inhibitor approved by FDA for the treatment of advanced EOC in patients who carry deleterious BRCA mutations and have received prior-line chemotherapy. It was later approved by FDA as maintenance therapy for patients with recurrent EOC regardless of BRCA mutations. With subsequent FDA approvals of two other PARP inhibitors rucaparib (Rubraca) and niraparib (Zejula), PARP inhibitors embody a promising class of agents for targeted EOC therapy. PARP inhibitors exploit synthetic lethality to target ovarian cancer with defects in homologous recombination repair (HRR) [9]. Because BRCA1 and BRCA2 proteins are critical components of the HRR pathway for DNA double strand breaks (DSBs), hereditary BRCA1 and BRCA2 mutations render EOC hypersensitive to DNA-damaging and PARP inhibitor therapy [10–12]. In clinical studies, EOC patients with hereditary BRCA mutations exhibit favorable responses to olaparib compared with patients without the mutations [13–15]. Similar clinical findings have been observed with rucaparib in which platinum-sensitive or BRCA-mutated EOC patients have a greater objective response rate and longer progression-free survival than platinum-resistant/refractory EOC patients [16, 17].

Despite the promising clinical activity of PARP inhibitors, the effectiveness of PARP inhibitors may be limited to certain patient populations. There is only a small subset (~15%) of EOC cases are BRCA-mutated [18]. It is estimated that up to 50% of high-grade serous EOC exhibit a phenotype of defective HRR without BRCA mutations, known as BRCAness [19]. However, the remaining considerable portion of EOC, both primary and recurrent, are intrinsically refractory or eventually acquire resistance to PARP inhibitors and platinum therapy. Reversion of mutated BRCA genes and restoration of HRR function have been identified in both preclinical and clinical studies of EOC with acquired resistance to platinum and PARP inhibitors [20–22]. Therefore, translational approaches are needed to overcome the limitation of PARP inhibitors for the treatment of resistant disease, as PARP inhibitors become a mainstay of therapeutic regimens for EOC.

Cediranib is a small molecule inhibitor of the tyrosine kinase of vascular endothelial growth factor (VEGF) receptor 1, 2, and 3. It blocks VEGFR signaling thereby exhibiting anti-angiogenic and vascular-normalizing activities against tumor growth [23]. The combination of cediranib and olaparib has been reported to significantly improve progression-free survival in patients with recurrent platinum-sensitive EOC, compared with patients treated with olaparib alone [24]. While the exact mechanisms by which cediranib enhances the anti-cancer activity of olaparib remains to be delineated, some plausible explanations, including down-regulation of HRR genes, suppression of cancer stemness, normalization of tumor vasculature for enhanced drug delivery, and inhibition of ascites development, have been postulated [24–27]. Nevertheless, cediranib is recently reported to augment the clinical efficacy of PARP inhibitor therapy for platinum-sensitive and platinum-resistant recurrent EOC regardless of BRCA status [28].

Triapine (3-aminopyridine-2-carboxaldehyde thiosemicarbazone) is the most potent small molecule inhibitor of ribonucleotide reductase (RNR) identified among a large number of thiosemicarbazones designed and synthesized in our laboratory [29–31]. Originally designed as a single anti-cancer agent, triapine was chosen based on its superb ability to inhibit DNA synthesis and cellular proliferation 1,000 times more potently than the clinically used RNR inhibitor hydroxyurea [29, 32]. Treatment of cells with triapine leads to depletion of dNTPs and stalls DNA replication [33, 34]. Preclinical and clinical studies demonstrate that triapine is used efficaciously as a chemo- and radio-sensitizing agent to enhance the anticancer activity of DNA damaging agents and radiation [32, 35–38]. With an impressive clinical response rate in phase I/II studies [35, 39, 40], triapine in combination with cisplatin and radiation therapy is currently under randomized phase II clinical trials for the treatment of advanced cervical cancers (NCT02466971). Clinically, triapine is very tolerable to patients but the notable side effects of methemoglobinemia and dyspnea associated with its strong iron-chelation property [41] are of concern.

Our mechanistic studies have previously elucidated that triapine impairs HRR thereby sensitizing BRCA-wild type EOC cells to PARP inhibitors and topoisomerase II inhibitors [37]. Furthermore, we have demonstrated that triapine augments the effects of platinum-based combination therapy to delay the growth of BRCA-wild type EOC xenografts in mice [38]. Given that the combination of olaparib and cediranib shows promising clinical activity against EOC, the rational combination of triapine, olaparib, and cediranib to treat BRCA-wild type and PARP inhibitor-resistant EOC was conceptualized and evaluated in our xenograft mouse models. The molecular mechanism of cediranib to enhance the efficacy of this combination therapy in vitro was also investigated and identified.

## Materials and methods

### Cell lines and chemicals

SKOV3 ovarian cancer cell line was grown in McCoys 5A medium supplemented with 10% FBS and penicillin-streptomycin antibiotics. OVCAR3 ovarian cancer cell line was grown in RPMI medium supplemented with 20% FBS and penicillin-streptomycin antibiotics. Both SKOV3 and OVCAR3 cell lines were purchased from ATCC (Manassas, VA). PEO1 and PEO4 ovarian cancer cell lines were grown in DMEM medium supplemented with 10% FBS and penicillin-streptomycin antibiotics. PEO1 and PEO4 cell lines were provided by Dr. Peter Glazer (Yale University) and confirmed by short-tandem repeat (STR) analysis (Promega-ATCC). Ascites-derived PEO1ip and PEO4ip cell lines were also authenticated by STR analysis (Yale DNA Analysis Facility on Science Hill). Olaparib and cediranib were purchased from Selleck (Houston, TX). Triapine (3-aminopyridine-2-carboxaldehyde- thiosemicarbazone) isethionate were synthesized in our laboratory as previously described [30] and custom-synthesized by Tocris-R&D Systems (Minneapolis, MN).

### Tumor xenografts and drug treatment

The Yale University Institutional Animal Care and Use Committee approved the protocol (IACUC# 2015–20038) for the in vivo animal studies in compliance with the US Public Health Policy on Humane Care and Use of Laboratory Animals. Yale University is registered as a research facility with the United States Department of Agriculture, License and Registration number 16-R-0001 The School of Medicine is fully accredited by the American Association for Accreditation of Laboratory Animal Care (AAALAC). An Animal Welfare Assurance (D16-00146) is on file with OLAW-NIH; effective July 25, 2016.

Five to six weeks old female athymic Nude-Foxn1^nu^ mice and SCID-Beige mice were purchased from Envigo (Indianapolis, IN). For s.c. tumor xenograft experiments, SKOV3 or OVCAR3 cells suspended in 100 μl serum-free medium mixed with 50 μl Matrigel (BD Biosciences, Franklin Lakes, NJ) were implanted s.c. in the right dorsal medial area (3.6–10 x 10^6^ cells per mouse). Mice were randomly assigned to treatment groups, each of which consists of 3–5 mice. Therapy was initiated 3–10 days after implantation when tumors were approximately 50–100 mm^3^ in volume. For i.p. tumor xenograft experiments, PEO4ip cells suspended in 100 μl serum-free medium were implanted i.p. (10 x 10^6^ cells per mouse). Mice were randomly assigned to treatment groups and therapy was initiated 1–3 days after implantation. The control group of mice received i.p. treatment with vehicle (2% DMSO) and treatment groups of mice received i.p. treatment with single, double, and triple drug combinations once daily for 5 consecutive days per week. The size of s.c. tumor xenografts was measured three times per week using a digital caliper. Tumor volumes were calculated using the formula: length x width^2^/2. Body weights of mice were measured on every treatment day before administration and on the same schedule as tumor measurements during the treatment period.

The progression of PEO1ip was evaluated by the body condition score (BCS). The endpoint of survival was met when BCS2 (underconditioned) reached. BCS is based on the guideline for evaluating the overall health condition of animals [42] and for determining the endpoint for mice involved in abdominal tumor studies [43]. The progression of PEO4ip tumor xenografts was evaluated by the size of abdominal circumference using a ruler. A 50% increase in the abdominal circumference when BCS2 typically reached, was defined as the endpoint of survival. In contrast, mice without tumor implantation exhibited only 17% increase in abdominal circumference and maintained optimal BCS3 (well-conditioned) at the time when vehicle-treated PEO4ip-bearing mice reached a 50% increase in the abdominal circumference and BCS2.

### Establishment of intraperitoneal PEO1 and PEO4 cell lines

Because the PEO1 and PEO4 cell lines were poorly tumorigenic in immunodeficient mice, serial in vitro-in vivo transplantations of PEO1 and PEO4 cells were carried out. Ten x 10^6^ PEO1 and PEO4 cells were injected intraperitoneally (i.p.) into NOD-SCID and SCID-Beige mice, respectively. After about 140 days, mice developed ascites which was collected and grown in culture with DMEM medium with 10% FBS to establish the PEO1ip and PEO4ip cell lines. PEO1ip and PEO4ip cells were confirmed identical to PEO1 and PEO4 cells, respectively, by STR genotyping (DNA Analysis Facility at Science Hill, Yale University). Both cell lines were injected i.p. into SCID-Beige mice. PEO1ip cells caused wide spread organ metastases with little or no ascites. PEO1ip-bearing mice exhibited reduced activity and body weight due to cachexia about 60–70 days. In contrast, the xenografts of PEO4ip exhibited peritoneal progression as evidenced by ascitic development and abdominal distension. The abdominal circumference of PEO4ip-bearing mice reached a 50% increase about 60–70 days. PEO1ip and PEO4ip xenografts exhibited peritoneal progression with more than 90 and 95% penetrance, respectively, in SCID-Beige mice.

### Tumor histology

s.c. tumors were excised immediately after euthanasia and fixed with 10% formalin for 72 hr. Fixed tumor tissue was paraffin-embedded and sectioned for haematoxylin and eosin (H&E) staining. Ascites was obtained from mice immediately after euthanasia and mixed with Shandon Cytorich Red Collection Fluid (Thermo Fisher Scientific, Waltham, MA). Following centrifugation, a small fraction of cell pellets was processed by ThinPrep processor (Hologic, Marlborough, MA) to prepare ThinPrep slides with Pap stain.

### Human to mouse dose conversion

The dose conversion from human to mice was based on the guideline published by the National Cancer Institute [44]. The mouse dose equivalency was obtained by the multiplying human dose by the factor of 12. The assumption of the human average body surface area was 1.6 m^2^. The conversion of surface area to weight was calculated by multiplying by the km factor of 37. The triapine dose was further converted to the triapine isethionate equivalency by multiplying by the factor of 1.65. The molecular weight of triapine is 195.2 and the molecular weight of triapine isethionate is 321.4.

### Statistical analysis

Statistical analysis was performed using the Prism 7.01 software (GraphPad, La Jolla, CA). Data were expressed as means with standard error (SE) in control and treatment groups. For s.c. tumor xenograft models, comparisons between control and individual treatment groups and multiple pairwise comparison were made by the Wilcoxon matched-pairs signed test (tumor growth curve) or the one way ANOVA with the Dunnett’s multiple comparison test (tumor weight). For i.p. tumor xenograft models, comparisons were carried out by the one-way ANOVA with the Dunnett’s multiple comparison test compared with the control (abdominal circumference), or the Mantel-Cox test (Kaplan-Meier survival curve) compared with the control and between treatment groups. *p* values were presented at the significance level *p*<0.05, *p*<0.01, or p<0.001.

### Western blot analysis

The methodology was described previously [37, 38]. The BRCA2 antibody that detects both full-length and truncated forms of BRCA2 was purchased from Bethyl (Montgomery, TX). The anti-HSC70 antibody was purchased from Santa Cruz (Santa Cruz, CA). Phospho-AKT (Ser473), AKT, mTOR, phospho-S6 (Ser235/236), S6, phospho-FoxO1 (Ser248), cyclin A, phospho-Rb (Ser780), Rb, and p27 Kip1 antibodies were purchased from Cell Signaling (Danvers, MA).

### MTS cytotoxicity assays

The assay was performed as described previously [33, 45]. PEO1 and PEO4 cells were plated into 96 well plates for 24 hr. Thereafter, cells were pre-treated with or without cediranib, triapine, or both drugs for 1 hr and then treated with various concentrations of olaparib for 72 hr. MTS reagent (Promega; Madison, WI) was added to wells for 2 hr and the plates were read by a plate reader. Values of absorbance was calculated to determine percent cell survival relative to vehicle-treated controls.

### Excess over bliss (EOB) analysis

Effects of two drugs in combination on cell survival were determined by EOB based on the principle of Bliss independence [46]. The value of EOB was calculated by the formula: EOB = (Ev-Cv) x 100, where Ev is the experimental value and Cv is the calculated value. Cv = Ea+Eb-Ea x Eb, where Ea is the fraction affected by drug a and Eb is the fraction affected by drug b. EOB > 0 indicates synergy, EOB = 0 indicates additivity, and EOB < 0 indicates antagonism.

### Caspase 3/7 assays

The assay was performed as described previously [47]. At the end of 24 hr and 48hr drug treatment, cells were lysed with the lysis buffer (PBS, 1% NP40, 0.1% SDS). Ten μl of lysate was incubated with Caspase-Glo 3/7 Assay reagent (Promega) at room temperature for 1 h and subsequently luminescence was measured with a luminometer (Turner Designs/Promega). Total protein concentration of cell lysates was determined as described above. Caspase 3/7 activity [luminescence units (RLU)] was normalized to protein concentration and expressed as the fold change in apoptosis with respect to the vehicle-treated control in each cell line.

### Cell cycle analysis

During the final hour of drug treatment, cells were labeled with 10 μM EdU (5-ethynyl-2' -deoxyuridine). Cells were then collected, fixed, permeabilized for detection of S phase population using Click-iT EdU Alexa Fluor 488 Flow Cytometry Assay Kit (Thermo Fisher Scientific), followed by counterstaining of DNA with 7-aminoactinomycin D (7-AAD) (BD Biosciences). Bivariate analysis of EdU incorporation and DNA content was performed by flow cytometry using LSRII flow cytometer (BD Biosciences) and FlowJo software (FlowJo LLC, Ashland, OR). Cell populations of G1, S, and G2/M phases were gated to determine the percentage of cells in each population. Statistical analysis was performed using the Student’s t-test.

## Results

### The combination of triapine, olaparib, and cediranib suppressed BRCA-wild type EOC tumor growth in the s.c. xenograft model

We have previously demonstrated that triapine impairs HRR and renders BRCA-wild type EOC cells sensitive to olaparib in culture [37]. We sought to evaluate the efficacy of the combination of triapine and olaparib to treat BRCA-wild type EOC in xenograft mouse model. Given that cediranib augments the activity of olaparib to treat recurrent platinum-sensitive EOC in patients [24], we also evaluated whether addition of cediranib furthered the efficacy of the olaparib-triapine combination. Two BRCA-wild type EOC cell lines SKOV3 and OVCAR3 [48] were used for the s.c. xenograft mouse model.

**Fig 1:**
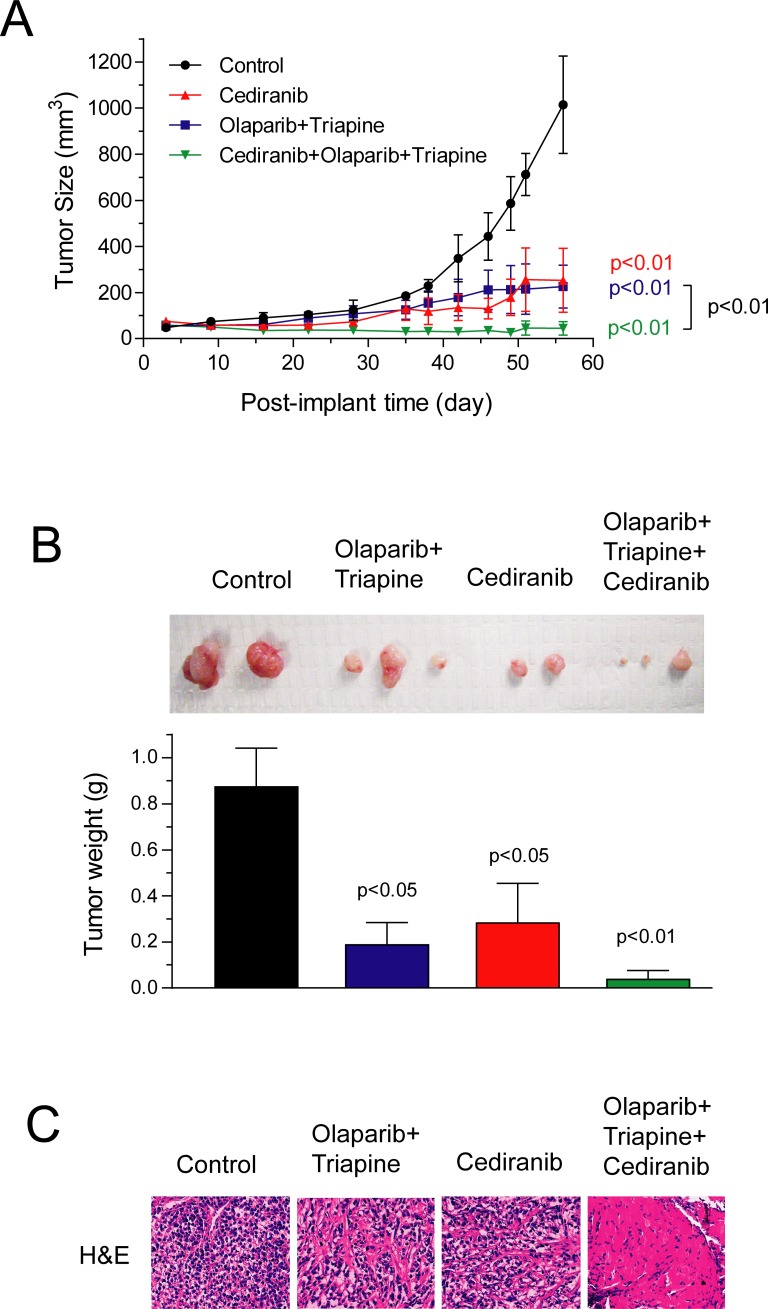
The effects of olaparib, triapine, and cediranib combinations on the growth of subcutaneous SKOV3 xenografts in mice. Athymic nude mice were inoculated s.c with SKOV3 cells. After 3 days, mice were randomly assigned to 4 groups (n = 3) and treated i.p. with vehicle, cediranib (0.75 mg/kg), the olaparib (50 mg/kg)-triapine (10 mg/kg) combination, and cediranib plus the olaparib-triapine combination daily for a continuous 6-week period (day 3 to 45). (A) Tumor size was measured every 2–3 days. Data are means ± SE. *p* values were determined by the Wilcoxon matched-pairs signed test compared with the control and between treatment groups. (B) Tumor tissue was excised from mice, photographed, and weighted in the end of the experiment. One largest tumor in control and cediranib groups is not shown. Data are means ± SE. *p* values were determined by the one-way ANOVA with the Dunnett’s multiple comparison test compared with the control. (C) Representative images of H&E-stained section of tumor tissue from respective treatment groups are shown.

Athymic nude mice inoculated s.c. with SKOV3 cells were treated daily with cediranib, the triapine-olaparib combination, and cediranib plus the triapine-olaparib combination for 6 weeks. The doses of cediranib, triapine, and olaparib were 0.75 mg/kg, 10 mg/kg, and 50 mg/kg, respectively. The tumor size was measured, and the body weight of mice was monitored over the course of drug treatment. We have previously shown that triapine at 10 mg/kg has no inhibitory effects on the growth of SKOV3 xenografts in mice [38]. Our preliminary studies also showed that olaparib alone at 50 mg/kg had no inhibitory effects on BRCA-wild type SKOV3 xenografts but caused marked suppression of the growth of BRCA1-knockdown SKOV3 xenografts (S1 Fig). In this study, the triapine-olaparib combination significantly suppressed the growth of s.c. SKOV3 xenografts (Fig 1A). Cediranib alone at 0.75 mg/kg also significantly suppressed the tumor growth. Remarkably, cediranib plus the triapine-olaparib combination achieved nearly complete suppression of tumor growth to the extent statistically greater than the triapine-olaparib combination (p<0.01). Furthermore, tumor tissue excised from mice in the end of the experiment exhibited a reduction in tumor weight in a manner similar to the endpoints of tumor growth curves (Fig 1B). H&E sections of SKOV3 tumor tissue showed that the combination of triapine, olaparib, and cediranib led to a pronounced shrinkage and disappearance of nuclei in sharp contrast to that of control, cediranib, and the triapine-olapaib combination (Fig 1C).

**Fig 2:**
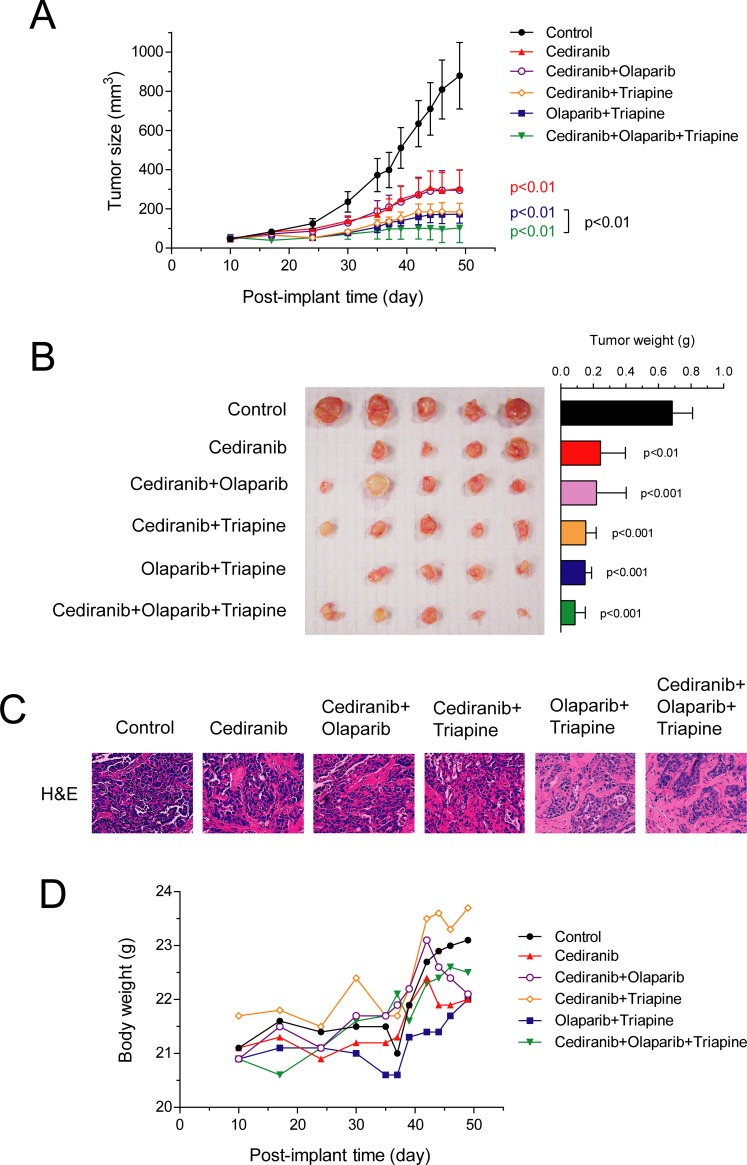
The effects of olaparib, triapine, and cediranib combinations on the growth of subcutaneous OVCAR3 xenografts in mice. Athymic nude mice were inoculated s.c with OVCAR3 cells. After 10 days, mice were randomly assigned to 6 groups (n = 5) and treated i.p. with vehicle, cediranib (0.75 mg/kg), olaparib (50 mg/kg) plus cediranib, triapine (10 mg/kg) plus cediranib, olaparib plus triapine, and olaparib plus triapine plus cediranib daily for consecutive 6 weeks (day 10 to 52). (A) Tumor size was measured every 2–3 days. Data are means ± SE. *p* values were determined by the Wilcoxon matched-pairs signed test compared with control and between groups. (B) Tumor tissue was excised from mice, photographed, and weighted in the end of the experiment. Data are means ± SE. *p* values were determined by the one-way ANOVA with the Dunnett’s multiple comparison test compared with the control. (C) Representative images of H&E-stained section of tumor tissue from each treatment group are shown. (D) The body weight of mice was measured every 2–3 days. The means of body weight for all treatment groups are shown.

Athymic nude mice inoculated s.c. with OVCAR3 cells were treated daily with cediranib, cediranib plus triapine, cediranib plus olaparib, the triapine-olaparib combination, and cediranib plus the triapine-olaparib combination for 6 weeks, at dose levels of all drugs identical to the SKOV3 xenograft experiment. Cediranib alone produced considerable suppression of the s.c. OVCAR3 tumor growth (Fig 2A). Cediranib plus olaparib yielded tumor growth inhibition not greater than cediranib alone. In contrast, cediranib plus triapine and the triapine-olaparib combinations resulted in somewhat greater growth inhibition. Consistent with the result of s.c. SKOV3 xenografts, cediranib plus the triapine-olaparib combination led to significantly enhanced inhibition of tumor growth compared with that caused by the triapine-olaparib combination (p<0.01). Tumor tissue excised from mice in the end of the experiment confirmed a reduction in tumor weight corresponding to the endpoints of tumor growth curves (Fig 2B). H&E section of OVCAR3 tumor tissue showed that the combination of triapine, olaparib, and cediranib resulted in reduced number of nuclei compared with that of control, cediranib and other double combinations (Fig 2C). Moreover, mice in all drug treatment groups exhibited no apparent reduction in body weight compared with control mice treated with vehicle (Fig 2D). These results collectively suggest that the combination of triapine, olaparib, and cediranib suppresses the growth of s.c BRCA-wild type EOC xenografts in nude mice. In addition, drug dosage and duration of the combination treatment are very tolerable to nude mice and result in no overt toxicity.

### The combination of triapine, olaparib, and cediranib delayed peritoneal progression of EOC and prolonged the survival of mice in the i.p. xenograft model

PEO4 cells are a high-grade EOC cell line derived from a patient at the second relapse following platinum-based chemotherapy. The patient who carried a hereditary BRCA2 mutation initially developed platinum-sensitive EOC but progressed to platinum-resistant disease at which PEO4 cells were obtained [49]. PEO4 cells have been confirmed to be platinum-resistant as a result of the reverted mutation of mutated BRCA2 gene to restore the function of the wild type gene [50]. Unfortunately, we found that PEO1 and PEO4 cells did not form s.c. tumors in most immunodeficient mice tested.

**Fig 3:**
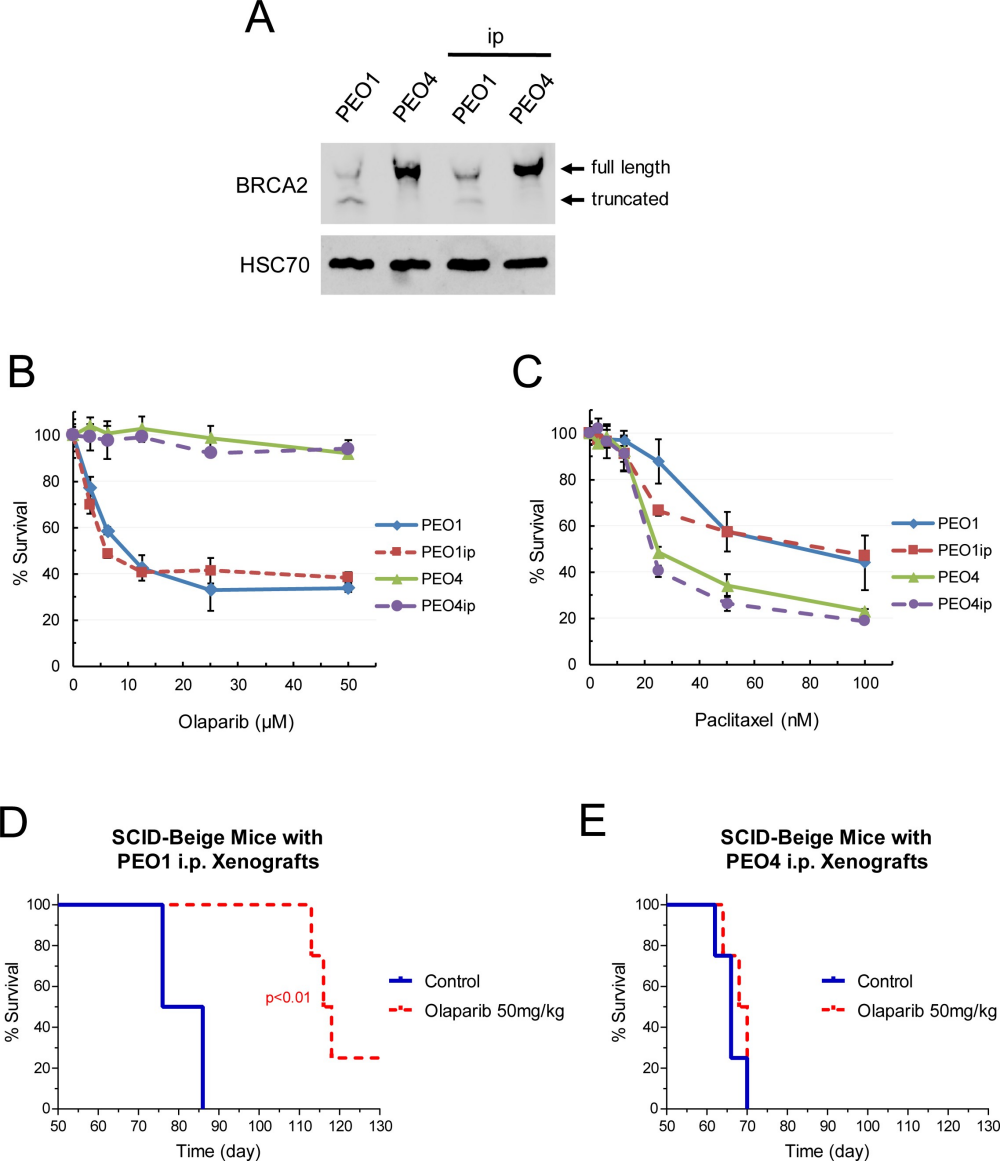
Characterization of intraperitoneal EOC cell lines and xenografts. (A) Expression of BRCA2 in PEO1/4 cells and PEO1/4ip cells. Total protein was isolated from cells and subjected to western blot analysis for BRCA2 protein. HSC70 protein was used as a loading control. BRCA2 wild type and mutant bands are shown. (B, C) Sensitivity of PEO1/4 cells and PEO1/4ip cells to olaparib and paclitaxel. Cells were treated with various concentrations of olaparib or paclitaxel for 72 hr. MTS cytotoxicity assay was performed to determine percent survival relative to vehicle-treated controls. Data are means ± SE. (D, E) SCID-Beige mice were inoculated i.p. with PEO1ip or PEO4ip cells. After 3 days, mice were randomly assigned to 2 groups (n = 4) and treated i.p. with vehicle and olaparib (50 mg/kg) daily for 6 weeks (day 3 to 45). The body condition score (BCS) of mice bearing PEO1ip xenografts was monitored and the abdominal circumference of mice bearing PEO4ip xenografts was measured every 2–3 days to determine the endpoint and the Kaplan-Meier survival curve. *p* values were determined by the Mantel-Cox test compared with the control.

To circumvent the problem and to enhance the ability of PEO1 and PEO4 cells to establish native peritoneal growth, we performed serial in vitro-in vivo transplantation of PEO1 and PEO4 cells. The resultant PEO1ip and PEO4ip cell lines demonstrated reliable peritoneal growth capabilities and a defined rate at which the survival endpoint reached (60–70 days). In additional to STR genotyping, the status of BRCA2 expression in PEO1ip and PEO4ip cells was determined by western blot analysis and confirmed identical to parental PEO1 and PEO4 cells, respectively (Fig 3A). The sensitivities of PEO1ip and PEO4ip cells to olaparib and paclitaxel were also comparable to parental PEO1 and PEO4 cells, respectively (Fig 3B and 3C). Paclitaxel was used to determine drug sensitivity that does not relate to DNA damage. These results suggest intact characteristics of BRCA2 and HRR status during the process of serial transplantation. Furthermore, the sensitivities of PEO1ip and PEO4ip xenografts to olaparib were determined in SCID-Beige mice. Mice implanted with PEO1ip or PEO4ip were treated with vehicle and olaparib (50 mg/kg) daily for 6 weeks. Treatment of mice with olaparib resulted in pronounced and significant prolongation of the survival time of mice bearing PEO1ip xenografts (p<0.01) but had no effects on the survival time of mice bearing PEO4ip xenografts (Fig 3D and 3E).

**Fig 4:**
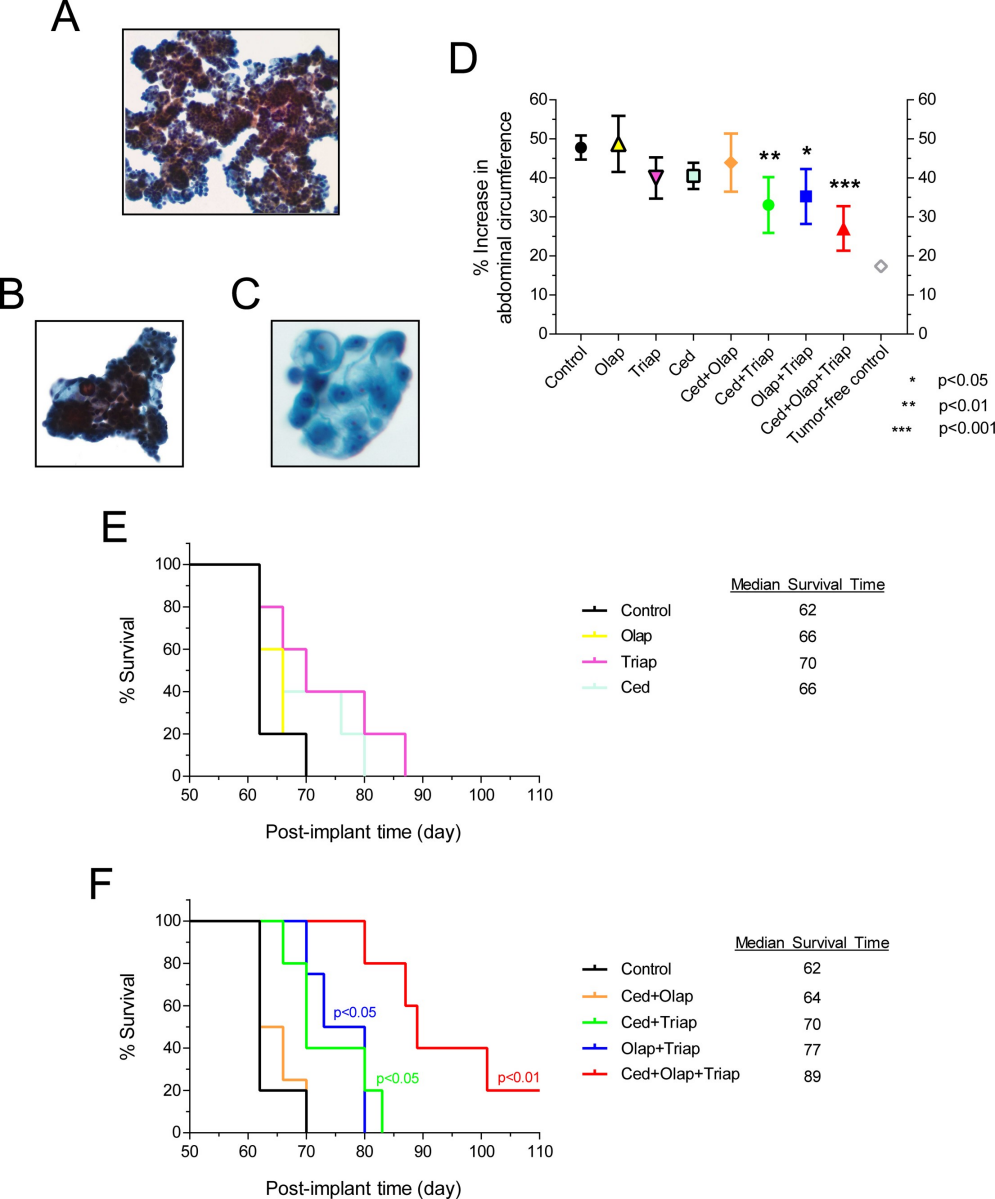
The effects of olaparib, triapine, and cediranib combinations on peritoneal progression of PEO4ip xenografts and the survival time of mice. SCID-Beige mice were inoculated i.p. with PEO4ip cells. After 3 days, mice were randomly assigned to 8 groups (n = 5) and treated i.p. with vehicle, single, double, and triple combinations consisting of cediranib (0.75 mg/kg), olaparib (50 mg/kg), and triapine (10 mg/kg) daily for two 3-week periods with a 2-week treatment-free period in between (day 3 to 24 and day 38 to 59). The abdominal circumference was measured and the body condition was monitored every 2–3 days. (A, B) Ascitic fluid smears on ThinPrep slides showed three-dimensional malignant cell clusters with papillary arrangement. (C) High-power microscopic view showed cells with cytological features consistent with ovarian high-grade serous carcinoma. (D) Peritoneal progression of PEO4ip xenografts leading to ascitic development was determined and expressed as a percent increase in abdominal circumference at day 62. Data are means ± SE. *p* values were determined by the one-way ANOVA with the Dunnett’s multiple comparison test compared with the control. Tumor-free control is the group of mice (n = 2) without implantation of PEO4ip cells. (E, F) The Kaplan-Meier survival curve was determined using a 50% increase in abdominal circumference and BCS2 as the endpoint. *p* values were determined by the Mantel-Cox test compared with the control. Olap, olaparib; Ced, cediranib; Triap, triapine.

**Fig 5:**
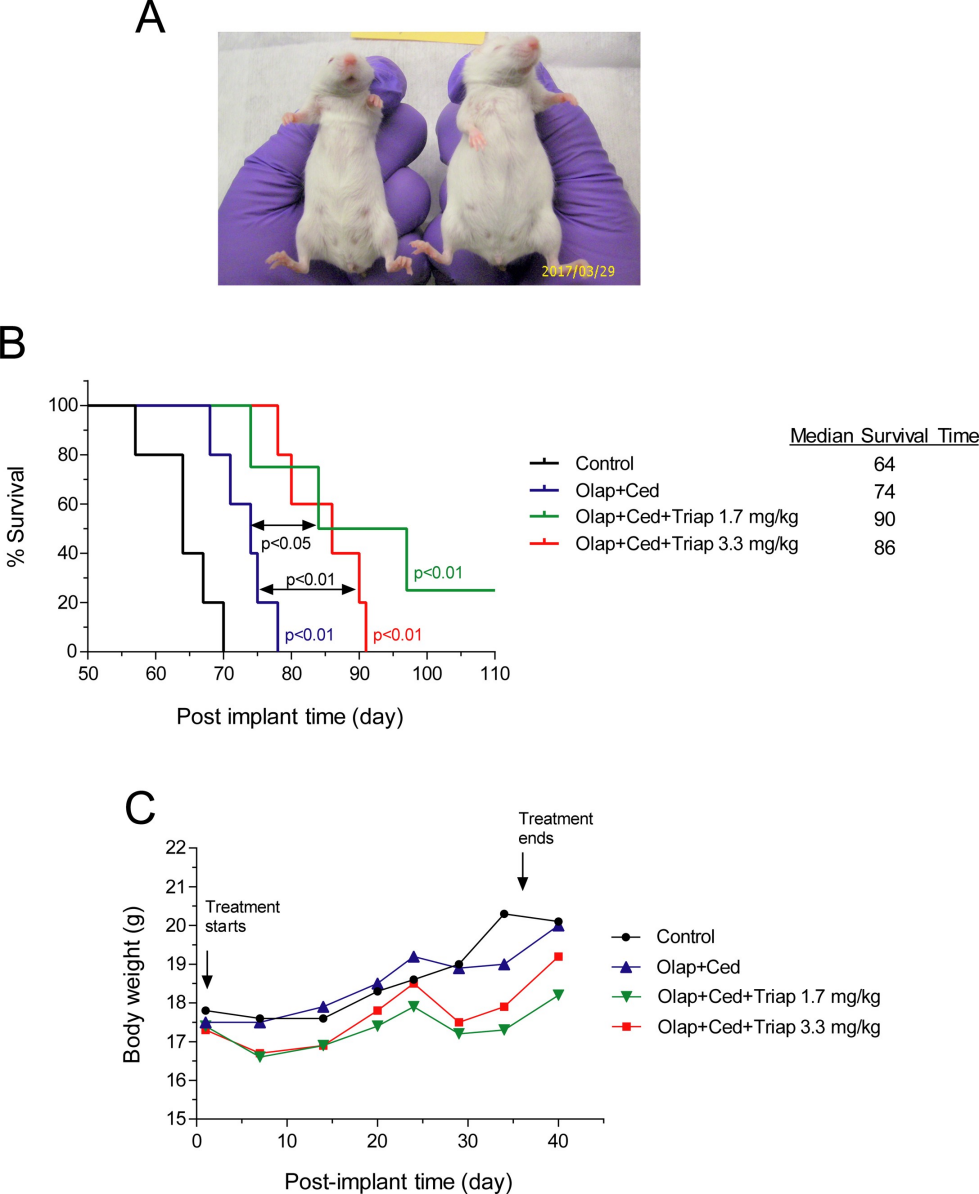
The effects of olaparib, triapine, and cediranib combinations at different dose levels on peritoneal progression of PEO4ip xenografts and the survival time of mice. SCID-Beige mice were inoculated i.p. with PEO4ip cells. After 1 day, mice were randomly assigned to 4 groups (n = 5) and treated i.p. with vehicle, the olaparib (135 mg/kg)-cediranib (5 mg/kg) combination, and the same combination with triapine at 1.7 mg/kg or 3.3 mg/kg daily for a continuous 5-week period (day 1 to 36). The abdominal circumference was measured and the body condition was monitored every 2–3 days. (A) The representative appearance of mice from control (right) and triapine plus olaparib plus cediranib (left) groups at day 62 are shown. (B) The Kaplan-Meier survival curve was determined using a 50% increase in abdominal circumference and the attainment of BCS2 as the endpoint. *p* values were determined by the Mantel-Cox test compared with the control and between treatment groups. (C) The body weight of mice was measured every 2–3 days. The means of body weight for treatment groups are shown. Olap, olaparib; Ced, cediranib; Triap, triapine.

The effects of drug combinations on PARP inhibitor-resistant PEO4ip cells were evaluated. Peritoneal progression of PEO1ip cells were effectively deterred by olaparib alone (Fig 3D and 3E) and thus were not used for the drug combination studies. PEO4ip cells were inoculated i.p. into SCID-Beige mice. Mice were then treated daily with vehicle, olaparib, triapine, cediranib, cediranib plus olaparib, cediranib plus triapine, olaparib plus triapine, and cediranib plus olaparib plus triapine for a total of 6 weeks, at dose levels of all drugs identical to SKOV3 and OVCAR3 xenograft studies. The triple combination group exhibited a small reduction (5–10%) of body weight after first three weeks. Thus, all treatments were paused for 2 weeks until the body weight of the triple combination group recovered. Subsequently all treatments resumed for additional three weeks. No loss of body weight was observed thereafter. After 7–8 weeks, peritoneal progression of PEO4ip xenografts became evident with the accumulation of malignant ascites, an increase in abdominal circumference, and the attainment of BCS2 level in all mice of the control group. To validate the progression of PEO4ip xenografts, ThinPrep slides of the peritoneal fluid from mice of the control group (upon reaching the endpoint) were made. Microscopic examination showed clusters of malignant epithelial cells. Tumor cells formed papillary or three-dimensional clusters with smooth border. Occasional psammoma bodies were seen (Fig 4A, 4B and 4C). Tumor cells had high nucleus-cytoplasm (N/C) ratios, prominent nucleoli, hyperchromatic nuclei and "lacy" cytoplasm with vacuolization (Fig 5C). The cytological features are consistent with metastatic ovarian high-grade serous carcinoma.

The effects of single, double, and triple combination of triapine, olaparib and cediranib on peritoneal progression of PEO4ip xenografts were determined. On the day of the endpoint of the control group, single drugs had no significant inhibitory effects on an increase in abdominal circumference compared with the vehicle control. In contrast, cediranib plus triapine, olaparib plus triapine, and cediranib plus olaparib plus triapine caused significant inhibition of an increase in abdominal circumference compared with the vehicle control (p<0.01, p<0.05, and p<0.001, respectively) (Fig 4D). Furthermore, the median survival time for treatment groups of vehicle, olaparib, triapine, cediranib, cediranib plus olaparib, cediranib plus triapine, olaparib plus triapine, and cediranib plus olaparib plus triapine was 62, 66, 70, 66, 64, 70, 77, and 89 days, respectively (Fig 4E and 4F). Statistical comparisons of survival curves showed that single drugs had no effects whereas the double combinations cediranib plus triapine and olaparib plus triapine significantly prolonged the survival time of mice (p<0.05). Furthermore, the triple combination of cediranib, olaparib, and triapine resulted in marked prolongation of survival time (p<0.01) significantly greater than any other treatment. These findings suggest that the combination of cediranib, triapine, and olaparib effectively deters the ascitic development and peritoneal progression of BRCA-wild type EOC xenografts.

The dose of triapine used in above studies was comparable to that of clinical trials in cervical cancer [35, 40]. However, the doses of olaparib and cediranib were considerably lower (one-third and one-eighth, respectively) than those used clinically in patients (Table 1). To mitigate the possible toxic effects of triapine on SCID-Beige mice and ensure the efficacy of the combination treatment, we further evaluated the effectiveness of reduced doses of triapine in combination with olaparib and cediranib at more clinically equivalent levels. Mice injected i.p. with PEO4ip cells were treated daily with the combination of olaparib (134 mg/kg) and cediranib (5 mg/kg) in the absence and presence of triapine at two reduced doses (1.7 and 3.3 mg/kg) for a consecutive 5-week period. All combinations deterred peritoneal progression of PEO4ip xenografts as manifested by a delayed increase in the abdominal circumference of mice (Fig 5A). At these dose levels, the combination of olaparib and cediranib produced a small but significant increase in the survival time of mice (p<0.01). Addition of triapine at 1.7 and 3.3 mg/kg to the combination furthered a significant prolongation of the survival time (p<0.05 and P<0.01, respectively) (Fig 5B). The median survival time for treatment groups of vehicle, cediranib plus olaparib, cediranib plus olaparib plus triapine (1.7 mg/kg), and cediranib plus olaparib plus triapine (3.3 mg/kg) was 64, 74, 90, and 86 days, respectively. The combinations did not produce evident outward toxicity as judged by body weight and condition of mice throughout the treatment period (Fig 5C). Ascites development did not interfere with body weight measurement, because a measurable increase in abdominal circumference occurred only after 50 days and was not detected in the end of treatment period (36 days). The results substantiate our findings that the combination of triapine, olaparib, and cediranib effectively deters ascitic development and peritoneal progression of BRCA-wild type EOC in mice. Adjustment of the ratio of triapine to the olaparib-cediranib combination is feasible to achieve maximal efficacy with minimal or no toxicity.

**Table 1:** Dose equivalency between human and mouse.

	Human Dose in Clinical Trials	Human Dose Converted to Mouse Dose	Mouse Dose Used in Experiments	
			Figs 1, 2 and 4	Fig 5
**Triapine**	25–50 mg/m^2^/2 days^a^	13–27 mg/kg/2 days^c^	10 mg/kg/day	1.7 and 3.3 mg/kg/day
**Olaparib**	400–800 mg/day^b^	81–162 mg/kg/day	50 mg/kg/day	134 mg/kg/day
**Cediranib**	30 mg/day^b^	6 mg/kg/day	0.75 mg/kg/day	5 mg/kg/day

^a^ Two hr intravenous infusion; Kunos CA et al. [35]

^b^ Oral tablets, twice daily for olaparib, once daily for cediranib; Liu JF et al, [24]

^c^ Triapine isethionate equivalency.

### Cediranib augmented the effects of olaparib and triapine on EOC cells in vitro

**Fig 6:**
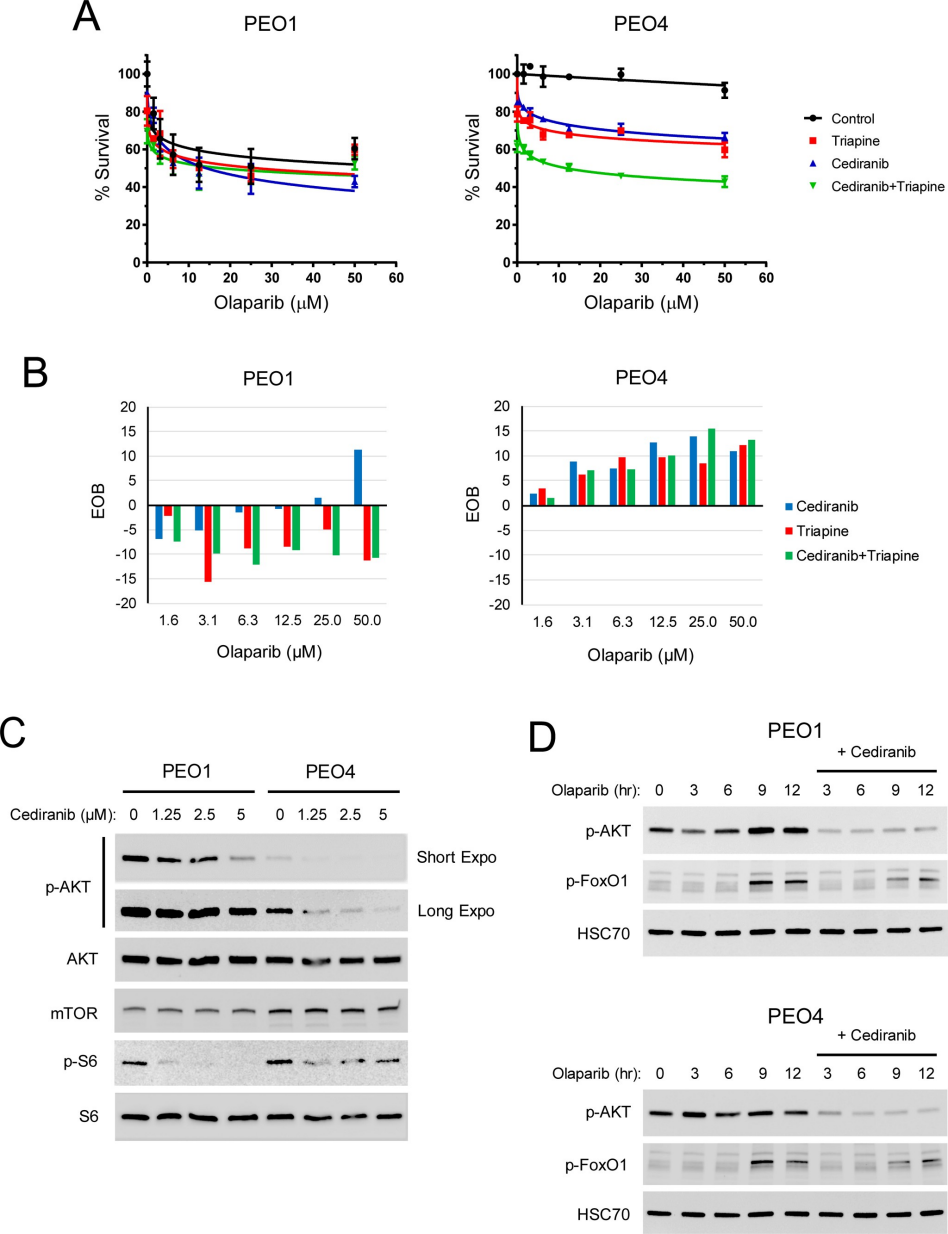
The effects of cediranib on AKT signaling and the sensitivity of BRCA2-wild type and mutated EOC cells to olaparib and triapine in vitro. PEO1 and PEO4 cells were treated with 1.25 μM cediranib, 0.75 μM triapine, or both drugs for 1 hr and then treated with various concentrations of olaparib for 72 hr. (A) MTS cytotoxicity assay was performed to determine percent survival relative to vehicle-treated controls. Data are means ± SD. (B) EOB was calculated to determine the effects of the combinations of cediranib, triapine, and olaparib on cell survival at all data points. EOB < 0 indicates antagonism. EOB = 0 indicates additivity. EOB > 0 indicates synergism. (C) PEO1 and PEO4 cells were treated with cediranib at 1.25, 2.5, and 5 μM for 24 hr. Western blot analysis was performed to determine the protein levels of phospho-AKT (Ser473), AKT, mTOR, phospho-S6 (Ser235/236), and S6. (D) PEO1 and PEO4 cells were serum-starved and then treated with 50 μM olaparib in the absence and presence of 5 μM cediranib for indicated time periods. Western blot analysis was performed to determine the protein levels of phospho-AKT (Ser473), phospho-FoxO1 (Ser248), and HSC70.

The anti-angiogenic activity of cediranib is widely attributed to tumor growth inhibition. Using the cell culture model, we sought to investigate whether cediranib treatment had a direct impact on the sensitivity of EOC cells to olaparib and triapine in the absence of angiogenic solid tumor environment. BRCA2-mutated PEO1 cells and BRCA-wild type PEO4 cells were treated with cediranib, triapine, or both drugs in combination with various concentrations of olaparib. In accord with HRR status, PEO1 cells were hypersensitive to olaparib whereas PEO4 cells exhibited pronounced olaparib resistance (Fig 6A). Treatment with cediranib or triapine sensitized PEO4 cells to increasing concentrations of olaparib. The combination of cediranib and triapine caused further sensitization of PEO4 cells to olaparib. In contrast, PEO1 cells were not further sensitized to olaparib by cediranib, triapine, or both drugs. Excess over Bliss (EOB) analysis confirmed that the combination of cediranib, triapine, and olaparib produced marked synergistic killing (EOC>0) of PEO4 cells while exhibiting mostly antagonistic effects (EOC<0) on PEO1 cells (Fig 6B). Notably, cediranib appeared to have synergistic effects on PEO1 cells toward the highest concentration of olaparib.

Activation of AKT promotes cell survival and proliferation, increases protein synthesis, and inhibits apoptosis [51]. It has been shown that autocrine and paracrine VEGF induces activation of AKT signaling in breast cancer [52, 53]. The effects of cediranib on AKT signaling in EOC cells were investigated. As we demonstrated previously [47], PEO1 cells exhibited a higher basal level of AKT phosphorylation or activity than PEO4 cells (Fig 6C). Regardless of this difference, cediranib markedly inhibited AKT phosphorylation in PEO1 and PEO4 cells in a dose-dependent manner (Fig 6C, short and long exposure). In addition, the phosphorylation of ribosomal protein S6, a readout of PI3K/AKT/mTOR signaling [54], was also attenuated by cediranib. We observed a lesser reduction in S6 phosphorylation in PEO4 cells than PEO1 cells. This phenomenon may result from the contribution of a higher mTOR level in PEO4 cells than PEO1 cells. AKT-independent regulation of mTOR signaling has been reported [55, 56].

In addition to growth factors, DNA damage has been shown to activate AKT presumably to maintain cell survival while DNA repair is in progress [57]. The effects of cediranib on olaparib-induced activation of AKT signaling were determined. Olaparib caused a gradual but pronounced increase in AKT phosphorylation, peaking at 9 hr and persisting at 12 hr (Fig 6D). PEO4 cells exhibited a relatively rapid but only modest increase in AKT phosphorylation peaking at 3 hr in response to olaparib treatment, indicative of functional HRR in action to mitigate DNA damage. The pro-survival property of AKT is mediated by phosphorylating and inhibiting FoxO1, a transcription factor that promotes apoptosis and inhibits cell cycle progression [58]. In line with increased AKT phosphorylation, inhibitory FoxO1 phosphorylation occurred at 9 and 12 hr treatment with olaparib in PEO1 and PEO4 cells (Fig 6D). Olaparib-induced phosphorylation of AKT and FoxO1 was considerably attenuated by cediranib.

**Fig 7:**
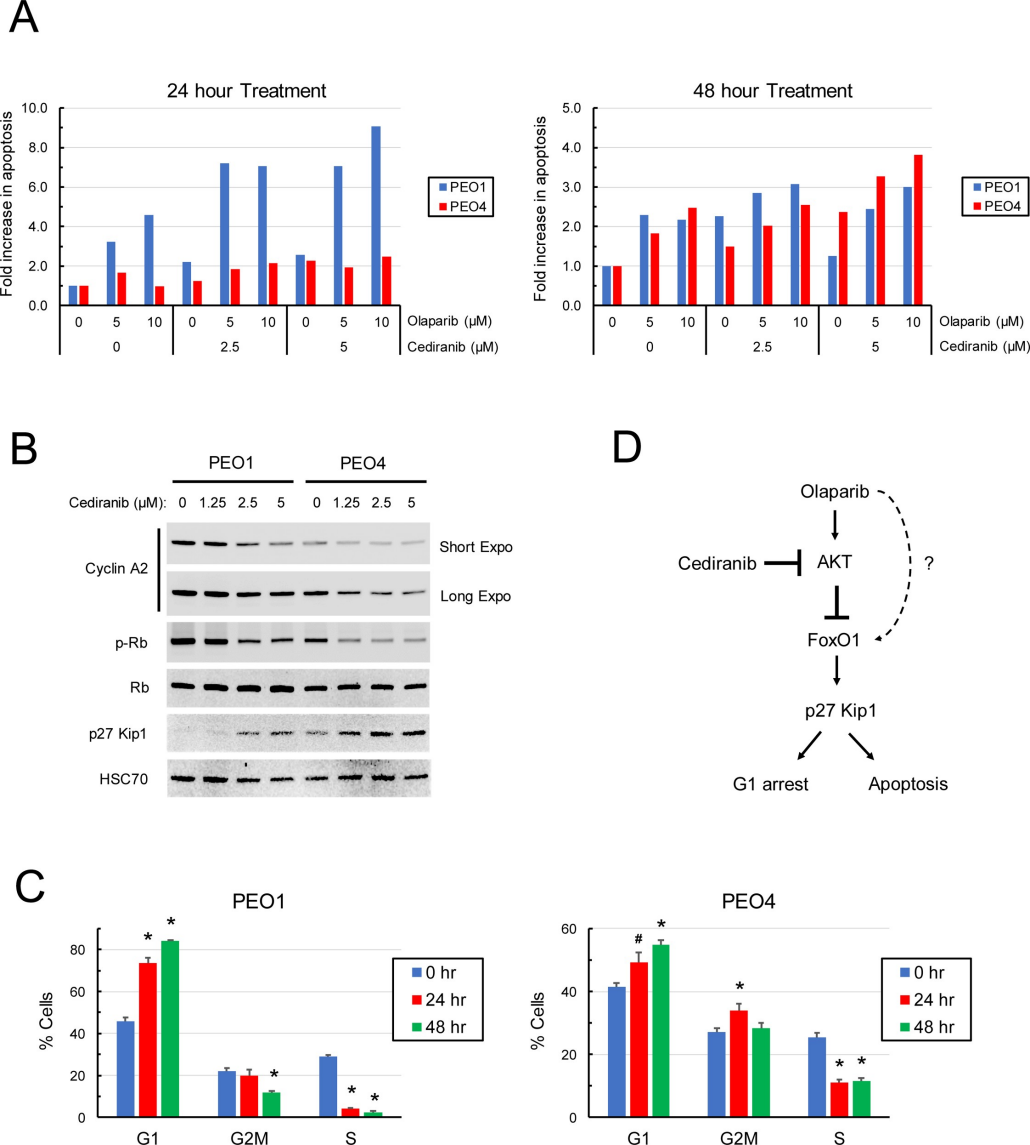
Effects of cediranib on olaparib-induced apoptosis and cell cycle progression in EOC cells. (A) PEO1 and PEO4 cells were pre-treated with 0, 2.5, and 5 μM cediranib for 1 hr and then treated with 0, 5, and 10 μM olaparib in the continuous presence of cediranib. After 24 and 48 hr of incubation, cells were lysed, and native protein was obtained to determine caspase 3/7 activity. The protein concentration of lysates was also determined to normalize the caspase 3/7 activity in each sample. The level of apoptosis is expressed as the fold change in the normalized caspase 3/7 activity with respect to the vehicle-treated control in each cell line. (B) PEO1 and PEO4 cells were treated with 0, 2.5, and 5 μM cediranib for 24 hr. Western blot analysis was performed to determine the protein levels of phospho-Rb (Ser780), cyclin A, p27 Kip1, and HSC70. (C) PEO1 and PEO4 cells were treated with 5 μM cediranib for 24 and 48 hr. Cells were pulse-treated with 10 μM EdU for 1 hr prior to flow cytometric analysis. G1, S, G2 populations were gated to show the percentage of cells in each phase. Data are means ± SD. *, *p* < 0.01; #, *p* < 0.05, compared with the 0 hr controls. (D) The AKT signaling pathway leads to blockade of the FoxO1-p27 Kip1-mediated apoptosis and G1 arrest. Cediranib inhibits olaparib-induced AKT activity, thereby enhancing the efficacy of olaparib (and triapine) against EOC. Olaparib also induces apoptosis possibly through FoxO1-p27 Kip1 and unknown mechanisms that remain to be determined.

The effects of cediranib on olaparib-induced apoptosis and cell cycle progression were investigated. Cediranib augmented the apoptotic effects of olaparib on PEO1 and PEO4 cells detected prominently at 24 hr and 48 hr, respectively, in a dose-dependent manner (Fig 7A). The earlier induction of olaparib-induced apoptosis in PEO1 cells than PEO4 cells was consistent with the fact that PEO1 cells lack HRR to mitigate the direct impacts of DNA damage. G1 to S phase transition is mediated by hyperphosphorylation of retinoblastoma protein (Rb) and release of the transcription factor E2F, which leads to an increase in cyclin A required for S phase progression [59]. Furthermore, FoxO1 induces up-regulation of p27 Kip1, a CDK-cyclin inhibitor that blocks G1 to S phase transition and concomitantly induces apoptosis [60]. AKT inhibition by cediranib led to a dose-dependent decrease in Rb phosphorylation, coinciding with a pronounced increase in p27 Kip1 and a reduction in the S phase cyclin A (Fig 7B), indicative of cell cycle arrest at the G1 phase in PEO1 and PEO4 cells. Cell cycle analysis substantiated that cediranib caused accumulation of the G1 phase population and a decrease in the S phase population of PEO1 and PEO4 cells (Fig 7C and S2 Fig). Collectively, our findings suggest that cediranib enhances the olaparib sensitivity of EOC cells by inhibiting AKT and consequently enhancing FoxO1-mediated apoptosis and cell cycle arrest. The cediranib-sensitive AKT signaling pathway involved in the modulation of the olaparib sensitivity of EOC cells is illustrated in Fig 7D.

## Discussion

We propose a rational and pragmatic approach to disrupt the HRR pathway for making PARP inhibitors effective beyond BRCA-mutated and BRCAness EOC. Besides proper patient selection based on BRCA mutations or HRR deficiency, PARP inhibitors are now used more broadly in hope of that patients with high-grade serous EOC, as much as 50%, exhibiting BRCAness would be benefited. However, the remaining patients still face uncertainty. Our findings will pave the way for overcoming this clinical challenge, providing therapeutic options for HRR-proficient EOC and EOC with acquired PARP inhibitor resistance predictively on the rise.

Given that RNR is a rate-limiting enzyme for dNTP synthesis and DNA replication, triapine was originally designed and developed as an anti-proliferative agent to treat cancers [30]. However, the most effective preclinical and clinical indication of triapine for cancer therapy has been proven in the setting of the combination with DNA damaging modalities [32, 35–38]. We have previously shown that triapine impairs CtIP-mediated DSB end resection and HRR in BRCA-wild type EOC cells [37, 38]. Using isogenic EOC cell lines, we have further demonstrated that triapine synergistically sensitizes BRCA-wild type EOC cells to olaparib, while having antagonistic or virtually no effects on the olaparib sensitivity of their BRCA-mutated counterparts in vitro (Fig 6A and 6B). Moreover, the effectiveness of the olaparib-triapine combination in vitro is substantiated by our BRCA-wild type EOC xenograft models (Figs 1, 2, 4 and 5). In support of this notion, the inhibitory effects of triapine or RNR inhibition on HRR have been independently reported [61, 62]. Collectively, these findings suggest that RNR inhibition induces HRR deficiency or BRCAness state which underlies the ability of triapine to sensitize BRCA-wild type cancer cells to PARP inhibitors, radiation, and other DNA damaging agents. Our present studies demonstrate a proof-of-principle approach to treat BRCA-wild type EOC with the combination of triapine and PARP inhibitor therapy.

The anticancer activity of cediranib has been widely attributed to its anti-angiogenic effects. In agreement with this notion, our results demonstrate that cediranib as a single drug is efficacious in our subcutaneous EOC models (Figs 1 and 2) as angiogenesis is critical for solid tumor growth. However, cediranib as a single drug does not significantly deter progression of EOC and prolong the survival time of mice in our peritoneal xenograft model (Fig 4E). In contrast to the subcutaneous xenograft model, peritoneal dissemination of PEO4ip xenografts manifests exclusively the growth of microscopic tumor cell clusters in massive ascitic fluids (Fig 4A and 4B). There is no visible solid tumor mass attached to organs or omentum in the peritoneal cavity, suggesting that angiogenesis contributes minimally to this mode of tumor progression. Furthermore, our positive results of in vitro cytotoxic assays for the olaparib, triapine, and cediranib combination support that cediranib enhances the efficacy of olaparib against EOC cells independent of its anti-angiogenic effects. Several lines of evidence indicate that EOC cells express both VEGFs and VEGFRs as paracrine and autocrine signaling for promoting survival [63–65]. We have, for the first time, presented the evidence that cediranib suppresses a series of AKT-mediated pro-survival and anti-apoptotic events in EOC cells (Figs 6 and 7). Therefore, our findings provide a potential explanation for why cediranib also enhances the efficacy of triapine and olaparib to deter the progression of EOC in peritoneal xenograft model. Nevertheless, we believe that the effectiveness of cediranib pivots on either its anti-angiogenic or anti-proliferative activities (but not mutually exclusive) dictated by the context of tumor microenvironment and the mode of tumor progression.

Our studies were initially carried out with olaparib at 50 mg/kg and cediranib at 0.75 mg/kg. These doses, when converted to human doses, appeared to be considerably lower than those used clinically (Table 1). We chose the dose of olaparib at 50 mg/kg primarily because a published study shows that daily and long-term administration at this dose effectively inhibits BRCA-mutated tumor growth in mice [66]. This dose level was also proven highly efficacious to prolong survival time of mice carrying BRCA2-mutated PEO4 xenografts (Fig 3D). Cediranib at 0.75 mg/kg is a sub-inhibitory dose for SKOV3 tumor growth based on a published study [23]. The mouse dose of triapine at 10 mg/kg used in our studies is similar to human does (25–50 mg/m^2^) in clinical trials of radio-chemotherapy for cervical and vaginal cancers [35, 40] (Table 1). Since the adverse events, such as hypertension, diarrhea, and nausea, caused by the olaparib-cediranib combination therapy at clinical doses have been reported in 70% of patients [24], dose reduction in olaparib and cediranib would be beneficial for patients when combined with triapine. Adverse events attributed to triapine treatment, notably methemoglobinemia associated with dyspnoea [41], has been reportedly low at 25 mg/m^2^ three times weekly for 5 weeks [35, 40].

We found that triapine at 10 mg/kg in combination with olaparib and cediranib appears to exhibit adverse effects after 3 week-treatment, as evidenced by the onset of 5–10% weight loss of SCID-Beige mice in this treatment group. After a 2-week treatment-free period for all treatment groups, the body weight of mice in the triple combination group recovered and all treatments resumed for additional 3 weeks. However, we did not observe any adverse effects caused by the triple combination in athymic nude mice throughout a continuous 6-week period. It is plausible that triapine at this dose level is less tolerable for SCID-beige mice due to their severe immunodeficiency or defects in non-homologous end-joining repair. Therefore, in the follow-up studies, we sought to reduce the dose of triapine to 1.7 and 3.3 mg/kg in combination with olaparib and cediranib at clinically-relevant doses (134 mg/kg and 5 mg/kg, respectively) (Table 1). Our results demonstrate that olaparib and cediranib combined with triapine at reduced doses effectively hinder peritoneal progression of BRCA-wild type EOC and prolong the survival of mice. A small fraction of the dose of triapine at 10 mg/kg is potent enough to augment the activity of the olaparib-cediranib combination doses used clinically. Notably continuous administration with this triple combination for 5 weeks did not cause any adverse effect on SCID-beige mice. These findings substantiate the efficacy of the combination of triapine, olaparib, and cediranib to treat BRCA-wild type EOC and the feasibility to minimize adverse events caused by the combination when applied to patients.

In summary, EOC is the most lethal gynecologic malignancy and there is an unmet need for the majority of patients who will not respond to PARP inhibitor therapy. Using triapine to create BRCAness state and cediranib to augment DNA damage-induced apoptosis, we have put forward a mechanism-based and proof-of-principle approach to leverage PARP inhibitor for the treatment of BRCA-wild type EOC. Our findings demonstrate its feasibility in vivo and hold tremendous promise for implementing this combination regimen to improve treatment outcomes of EOC in patients.

## Supporting information

S1 Fig Effects of olaparib on SKOV3 tumor xenografts in nude mice. Athymic nude mice were inoculated s.c. with 3.6 x 10^6^ SKOV3-NTC and SKOV3-BRCA1kd cell lines. Both cell lines were established and described previously [37]. NTC, non-target control. BRCA1kd, BRCA1-knockdown. After 5 days, mice (N = 3) were treated i.p. with vehicle or olaparib (50 mg/kg) once daily for a continuous 6-week period (day 5 to 47). Tumor size was measured as described in the Materials and Methods. Data are means ± SE. *p* values were determined by the Wilcoxon matched-pairs signed test compared with the control.
(TIF)

S2 Fig Cell cycle distribution of PEO1 and PEO4 cells treated with cediranib. PEO1 and PEO4 cells were treated with 5 μM cediranib for 24 and 48 hr. Cells were pulse-treated with 10 μM EdU for 1 hr prior to flow cytometric analysis. EdU (AlexaFluor 488-A) vs. 7-AAD (PerCP-Cy5-5-A) plots are shown. G1, S, G2 populations were gated to show the percentage of cells in each cell cycle phase.
(TIF)
