# Supplementary figures and images for: Combination of triapine, olaparib, and cediranib suppresses progression of BRCA-wild type and PARP inhibitor-resistant epithelial ovarian cancer
Source: PLoS One. 2018 Nov 16;13(11):e0207399. doi: 10.1371/journal.pone.0207399 (PMC6239325; doi:10.1371/journal.pone.0207399)

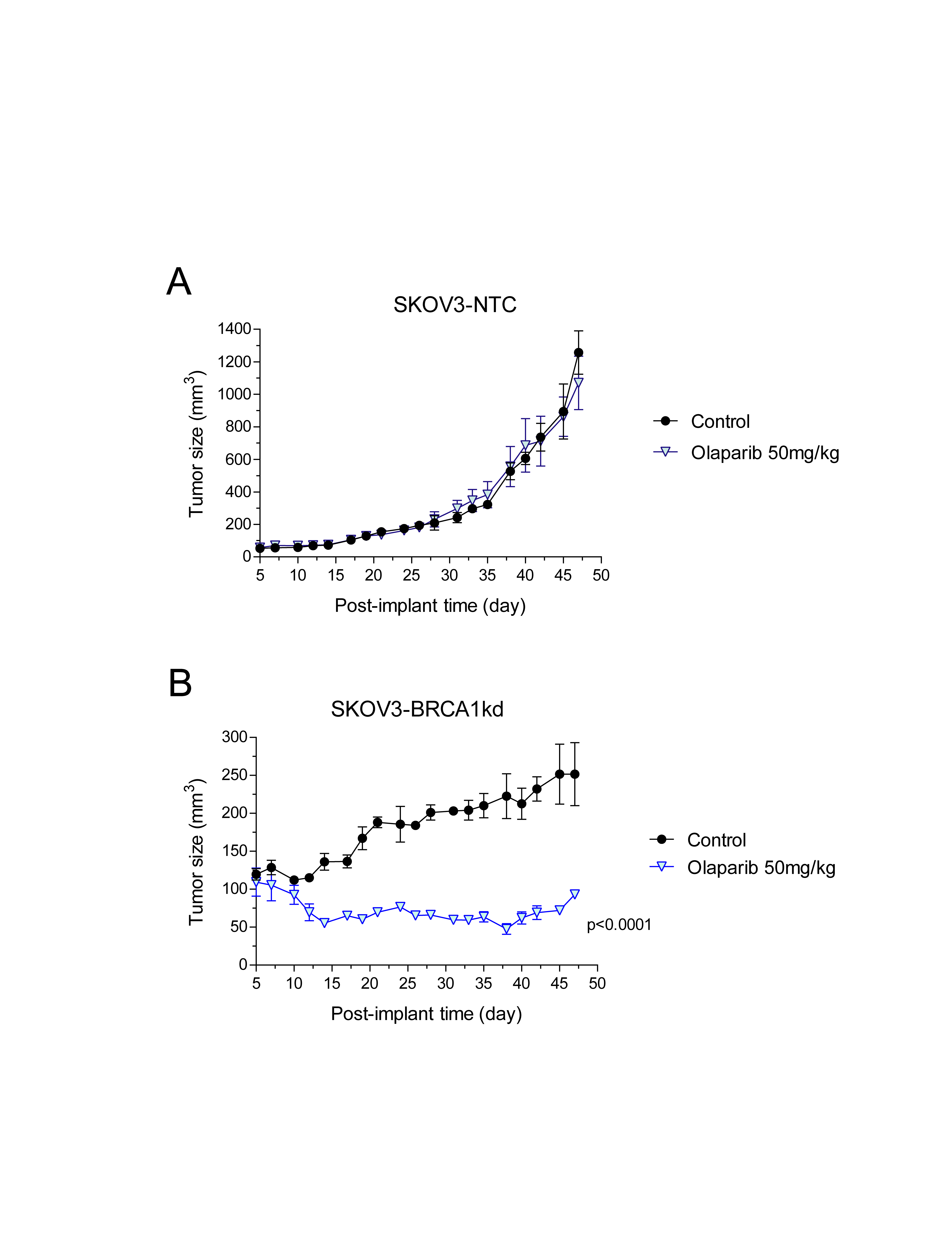

Supplement: S1 Fig — Athymic nude mice were inoculated s.c. with 3.6 x 10^6^ SKOV3-NTC and SKOV3-BRCA1kd cell lines. Both cell lines were established and described previously [37]. NTC, non-target control. BRCA1kd, BRCA1-knockdown. After 5 days, mice (N = 3) were treated i.p. with vehicle or olaparib (50 mg/kg) once daily for a continuous 6-week period (day 5 to 47). Tumor size was measured as described in the Materials and Methods. Data are means ± SE. p values were determined by the Wilcoxon matched-pairs signed test compared with the control. (TIF) [file pone.0207399.s001.tif]

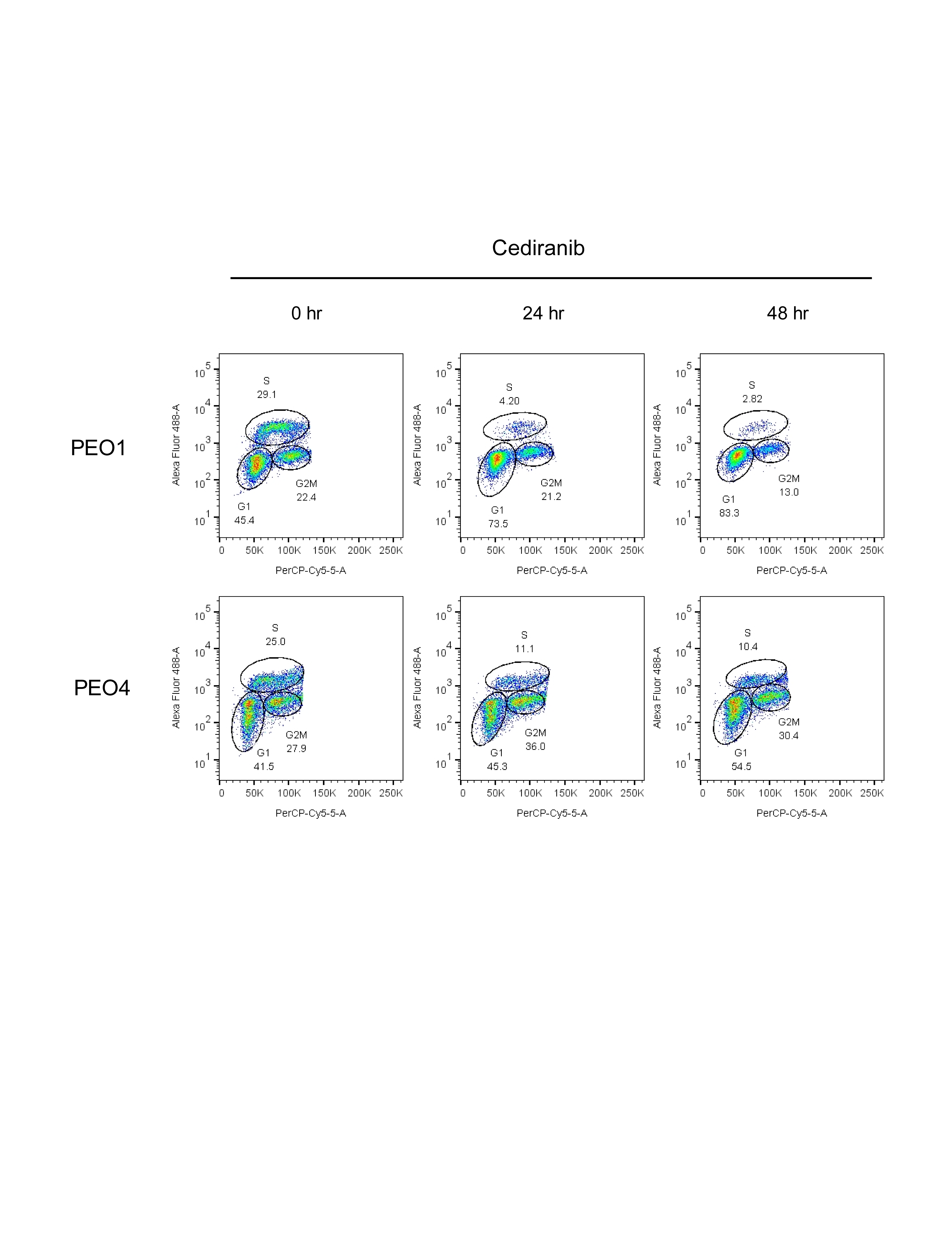

Supplement: S2 Fig — PEO1 and PEO4 cells were treated with 5 μM cediranib for 24 and 48 hr. Cells were pulse-treated with 10 μM EdU for 1 hr prior to flow cytometric analysis. EdU (AlexaFluor 488-A) vs. 7-AAD (PerCP-Cy5-5-A) plots are shown. G1, S, G2 populations were gated to show the percentage of cells in each cell cycle phase. (TIF) [file pone.0207399.s002.tif]
